# Prey selection and dietary flexibility of three species of mammalian predator during an irruption of non-cyclic prey

**DOI:** 10.1098/rsos.170317

**Published:** 2017-09-13

**Authors:** Emma E. Spencer, Thomas M. Newsome, Christopher R. Dickman

**Affiliations:** 1 Desert Ecology Research Group, School of Life and Environmental Sciences, University of Sydney, Sydney, New South Wales 2006, Australia; 2 School of Life and Environmental Sciences, Deakin University (Burwood Campus), Geelong, Victoria 3125, Australia; 3 Global Trophic Cascades Program, Department of Forest Ecosystems and Society, Oregon State University, Corvallis, OR 97331, USA; 4 School of Environmental and Forest Sciences, University of Washington, Seattle, WA 98195, USA

**Keywords:** dietary shifts, prey irruptions, prey selection, feral cat, red fox, dingo

## Abstract

Predators often display dietary shifts in response to fluctuating prey in cyclic systems, but little is known about predator diets in systems that experience non-cyclic prey irruptions. We tracked dietary shifts by feral cats (*Felis catus*), red foxes (*Vulpes vulpes*) and dingoes (*Canis dingo*) through a non-cyclic irruption of small mammalian prey in the Simpson Desert, central Australia. We predicted that all three predators would alter their diets to varying degrees as small mammals declined post irruption, and to test our predictions we live-trapped small mammals through the irruption event and collected scats to track predator diets. Red foxes and dingoes included a broader variety of prey in their diets as small mammals declined. Feral cats did not exhibit a similar dietary shift, but did show variable use and selectivity of small mammal species through the irruption cycle. Results were largely consistent with prior studies that highlighted the opportunistic feeding habits of the red fox and dingo. They also, however, showed that feral cats may exhibit less dietary flexibility in response to small mammal irruptions, emphasizing the importance of tracking predator diets before, during and after irruption events.

## 1. Introduction

Fluctuations in prey abundance may induce changes in dietary selection and the rate of prey consumption by predators [1,2]. Such changes are largely dependent on the foraging strategies used by predators to maximize their net rate of energy intake in response to variable prey availabilities [3]. Some predators may respond to fluctuating prey populations by including a greater variety of food types in their diet and by selecting prey items according to their availability. Such ‘generalist’ predators often display large dietary shifts as prey populations fluctuate [4–6]. In contrast, ‘specialist’ predators may use efficient hunting behaviours and persist in eating preferred prey items, even when such prey becomes scarce [7,8]. Both predators, however, may respond numerically to declining prey populations by emigrating or perishing in the system [4–6]. Developing an understanding of how predators manage their foraging behaviour in environments that experience highly changeable prey densities is therefore important, because the dietary choices of predators influence the distribution, abundance and survival of their prey [9], and because such choices influence the survival and overall fitness of the predators themselves [7,10].

Mammalian predators often display dietary shifts when small mammals fluctuate between periods of extremely high and low abundance. In south-central Sweden red foxes (*Vulpes vulpes*) target microtine voles, but hunt larger roe deer fawns (*Capreolus capreolus*) when vole densities decline [7]. In northern Belarus, foxes diversify their diets to include birds and fruits in the warm season and carrion and several species of vole in winter, but focus almost solely on voles when they become abundant during outbreaks [11]. Some predators, however, do not display strong dietary variation in response to small mammal fluctuations. For instance, in Greenland, the stoat (*Mustela erminea*) exhibits high dietary preference for cyclically fluctuating collared lemming (*Dicrostonyx groenlandicus*), even as this prey reaches very low densities [12]. Gilg *et al*. [12] suggested it was the stoat's ability to make use of the burrow network connecting lemming winter nests that enabled it to persist in eating lemmings during the low phase of the prey cycle.

Much of the research investigating how predators respond to variations in prey has focused on systems characterized by regular, cyclic prey fluctuations. Comparatively little is known of how predators use prey in environments that experience non-cyclic irruptions of prey. In contrast to cyclic systems, prey that irrupt in non-cyclic systems may experience more extended population lows, termed ‘busts’, and less regular increases, termed ‘booms’, which are often driven by intense but sporadic weather events such as intermittent, heavy rainfall [13,14]. Non-cyclic systems are also characterized by greater numerical differences in prey numbers between the boom and bust stages [15]. Hence, predators must contend with more variable prey availability; greater dietary flexibility, for example, might be required for predators to persist in these systems. Consequently, resident predators in non-cyclic systems probably favour generalist feeding strategies and may also display greater dietary opportunism compared with animals in cyclic systems. Alternatively, as drought periods precede and follow large rainfall events in non-cyclic systems, prey densities outside of the irruption are typically very low, and therefore neither specialist nor generalist diet strategies may be favoured. Predators instead could rely on high mobility and migration rather than dietary flexibility to avoid death [16]. Disentangling the differential use of prey by predators in non-cyclic systems is important, particularly as predators may persist at high densities post-irruption [17] and form ‘predator pits’, in which *per capita* predation pressure on prey is elevated and declining prey populations incur substantial predation costs [18,19]. In some environments, these predation costs could be substantial, especially when introduced predators with highly efficient hunting strategies are present [20].

The Simpson Desert, in central Australia, provides an excellent arena in which to examine the dietary responses exhibited by predators to non-cyclic prey irruptions. The region experiences irruptions of small mammals that are driven by large but irregular rain events [13]. The region also contains both introduced and native predator species [21]. In particular, the introduced feral cat (*Felis catus*) and red fox, and the native dingo (*Canis dingo*), co-occur in the region. All three predators show at least some dietary flexibility, although each species exhibits differing dietary preferences largely based on the size-class of their respective prey [22–24], and the feral cat is known to specialize on certain prey in some instances [25]. Although the general dietary habits of the feral cat, red fox and dingo are well studied across Australia, their dietary responses to fluctuating prey remain largely unexplored. Developing research in this area is integral to understanding how fluctuating prey affects predators, and how predators may in turn influence prey populations and communities. Indeed, the feral cat and red fox have been linked to the wave of mammal extinctions that continue to occur in Australia even now [26].

Here, we investigate the different dietary strategies—as revealed by scat analysis—exhibited by the feral cat, red fox and dingo, in response to fluctuating small mammalian prey. We expected that each predator would use irrupting and non-irrupting prey in different ways according to their dietary preferences, and also that each would show strong dietary shifts in response to declines in small mammal numbers. Based on these expectations, we generated three specific hypotheses. First, we predicted that all three predators would shift to alternative prey types when small mammal populations declined, but that the feral cat would maintain the highest proportion of its preferred prey type in its diet (i.e. small mammals) based on its comparatively stronger preference for certain prey types [27]. Second, we predicted that dietary diversity would increase for all three predator species as small mammal populations declined, and that this increase would be most pronounced for the red fox and dingo due to their broader, more opportunistic diets in comparison to the feral cat [27–29]. Third, as not all small mammal species in our system are irruptive, we also predicted that predators would alter their diets within the small mammal prey type, and would display strongest preferences for specific small mammal species during the post-irruptive stage. Our third prediction is based on the expectation that as fewer small mammals are available before or after an irruption event, predators must be more selective in order to meet their energy demands. We use the results to discuss how the feral cat, red fox and dingo use fluctuating prey populations in non-cyclic systems, and comment on the potential effects of variable foraging strategies on the predator species and on potentially vulnerable prey in the system [30].

## 2. Results

### 2.1. Overall diet trends

**Figure 1.**
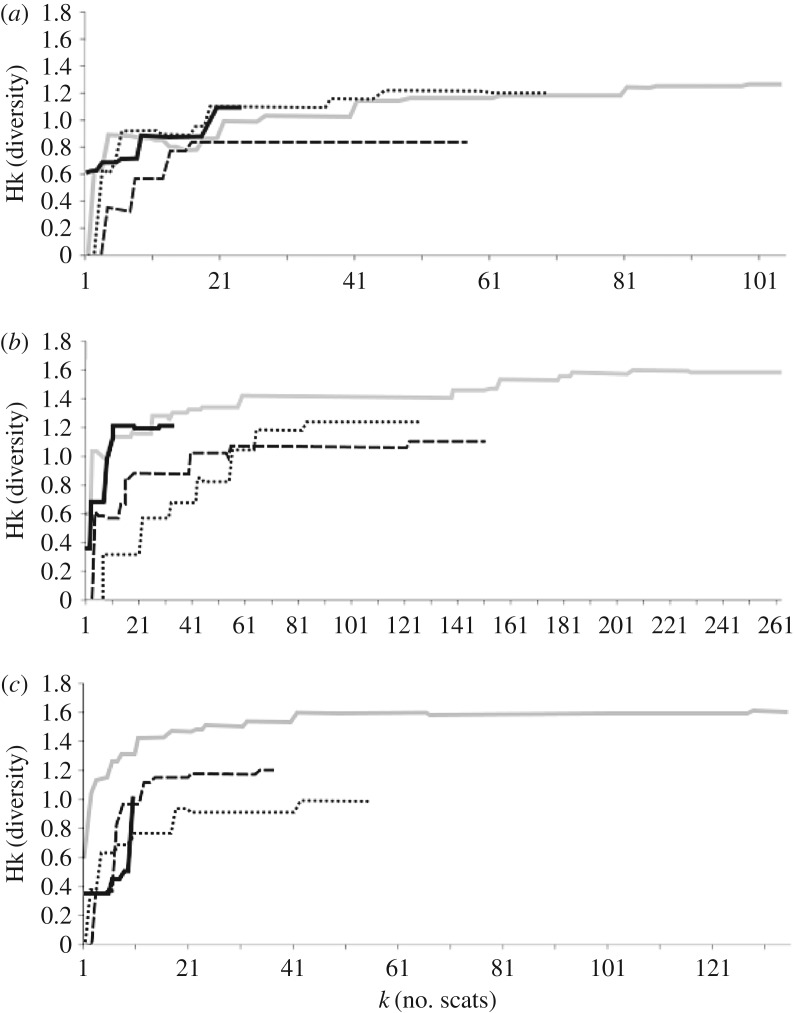
Cumulative dietary diversity of (*a*) feral cats (*Felis catus*), (*b*) red foxes (*Vulpes vulpes*) and (*c*) dingoes (*Canis dingo*), during the late bust (black line), boom (long dotted line), decline (short dotted line) and early bust (grey line) stages of the prey irruption cycle.

**Figure 2.**
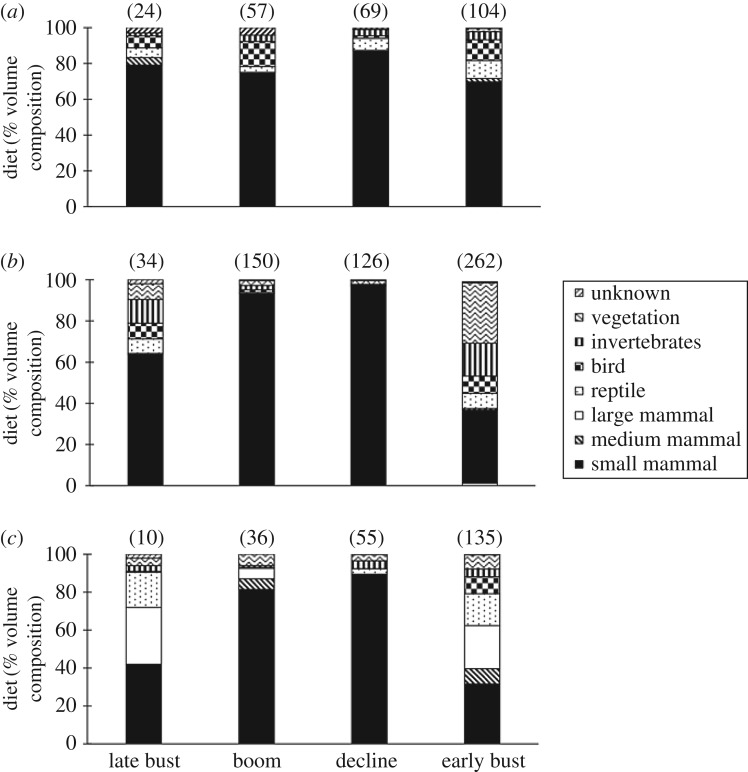
Diet of (*a*) feral cats, (*b*) red foxes and (*c*) dingoes shown as percentage volumes of major prey categories in scats. The numbers of scats analysed are shown in parentheses.

Throughout the study period, 254 feral cat scats, 572 red fox scats and 236 dingo scats were collected. Across each irruption stage, the cumulative diversity of prey items reached an asymptote for all predator species, except for the feral cat and dingo during the late bust stage, when only 24 and 10 scats were collected, respectively (figure 1). Over the four irruption stages, two large mammal and at least nine small mammal species were identified in scats (see electronic supplementary material, table S1). On the basis of percentage volume, small mammals, birds and reptiles were most commonly identified in feral cat scats. Small mammals, invertebrates, reptiles and vegetation were frequently identified in red fox scats, whereas dingo scats primarily included small mammals, large mammals and reptiles (figure 2).

### 2.2. Dietary shifts between prey types

Small mammals comprised the greatest volume of all prey types in each predator's scats, and declined markedly during the bust stage in scats of the red fox and dingo (figure 2). Dietary variation was confirmed in the PERMANOVA results (table 1), with differences between the irruption stages (d.f. = 3, *F* = 45.12, *p* < 0.001) and between the predator species (d.f. = 2, *F* = 11.39, *p* < 0.001); an interaction also occurred between the two factors (d.f. = 6, *F* = 10.001, *p* < 0.001). Pair-wise comparisons revealed consistent dietary differences between predator species during the irruption cycle (*p* < 0.038), but inconsistent differences within predator species at different times. For the dingo, pair-wise analysis revealed dietary differences between the boom and bust stages, and between the decline and bust stages, but for the feral cat the differences were between the boom and decline stages, and the decline and early bust stages (table 1). The red fox showed similar dietary disparity between stages to the dingo, but also displayed dietary differences between the late bust and early bust (table 1).

**Table 1.** PERMANOVA pair-wise comparisons of predator diets across the stages of a prey irruption cycle in the Simpson Desert, central Australia. Significant values (*p* < 0.05) are in italics.

predator species	groups	*t*	*p*
feral cat	late bust, boom	1.126	0.278
	late bust, decline	1.354	0.120
	late bust, early bust	1.089	0.317
	boom, decline	2.747	*0.003*
	boom, early bust	1.317	0.143
	decline, early bust	2.895	*0.001*
red fox	late bust, boom	5.735	*0.001*
	late bust, decline	6.730	*0.001*
	late bust, early bust	2.945	*0.001*
	boom, decline	1.404	0.132
	boom, early bust	10.515	*0.001*
	decline, early bust	10.257	*0.001*
dingo	late bust, boom	2.360	*0.008*
	late bust, decline	3.535	*0.001*
	late bust, early bust	0.800	0.644
	boom, decline	1.284	0.136
	boom, early bust	3.882	*0.001*
	decline, early bust	5.571	*0.001*

The high occurrence of small mammals in red fox and dingo scats during the boom and decline stages was the primary cause of dissimilarity in diet between the irruption stages (figure 2). Invertebrates and vegetation were also important to the red fox in the early bust and late bust stages, while reptiles and large mammals were more important in the diet of the dingo during these stages (figure 2). Small mammals were most important for the feral cat during all stages of the irruption cycle, although reptiles and invertebrates featured prominently in the early bust and birds in the boom and early bust stages (figure 2).

### 2.3. Variation in dietary diversity

**Figure 3.**
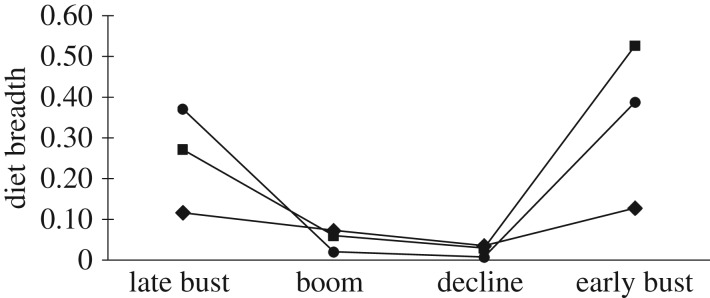
Dietary breadth indices for feral cats (diamond), red foxes (circle) and dingoes (square) during four stages of a prey irruption cycle in the Simpson Desert, central Australia.

All predators displayed the greatest dietary diversity during the early bust stage followed by the late bust stage for the feral cat and red fox, and lowest diversity during the boom or decline stages (figure 1). Over all stages, the feral cat's dietary diversity varied from 0.8 to 1.3, whereas the dingo and red fox showed greater diversity ranges (0.9–1.6 and 1.1–1.6, respectively; figure 1). These results were supported by Levins' index, which showed the highest dietary breadth for the dingo and red fox during the late bust and early bust stages and the lowest during the boom and decline stages (figure 3). The feral cat's dietary breadth exhibited little variation across the four stages. During the late bust stage, the red fox had the highest dietary breadth, and during the early bust stage the dingo had the highest breadth. The feral cat had the lowest dietary breadth in both the late and the early bust stages. Across the boom and decline stages, all predators had relatively similar dietary breadths (figure 3).

### 2.4. Dietary selection of small mammal species

Trapping between June 2008 and July 2013 yielded 3820 small mammal captures (see electronic supplementary material, table S2 and figure S1). Irruptive small mammal species included the spinifex hopping-mouse (*Notomys alexis*), the sandy inland mouse (*Pseudomys hermannsburgensis*), the long-haired rat (*Rattus villosissimus*) and the introduced house mouse (*Mus musculus*; electronic supplementary material, table S2). Long-haired rats and desert mice (*Pseudomys desertor*) were only captured during the boom and decline stages, as were more than 70% of all house mice, spinifex hopping-mice, wongai ningauis (*Ningaui ridei*) and sandy inland mice (see electronic supplementary material, table S2).

**Figure 4.**
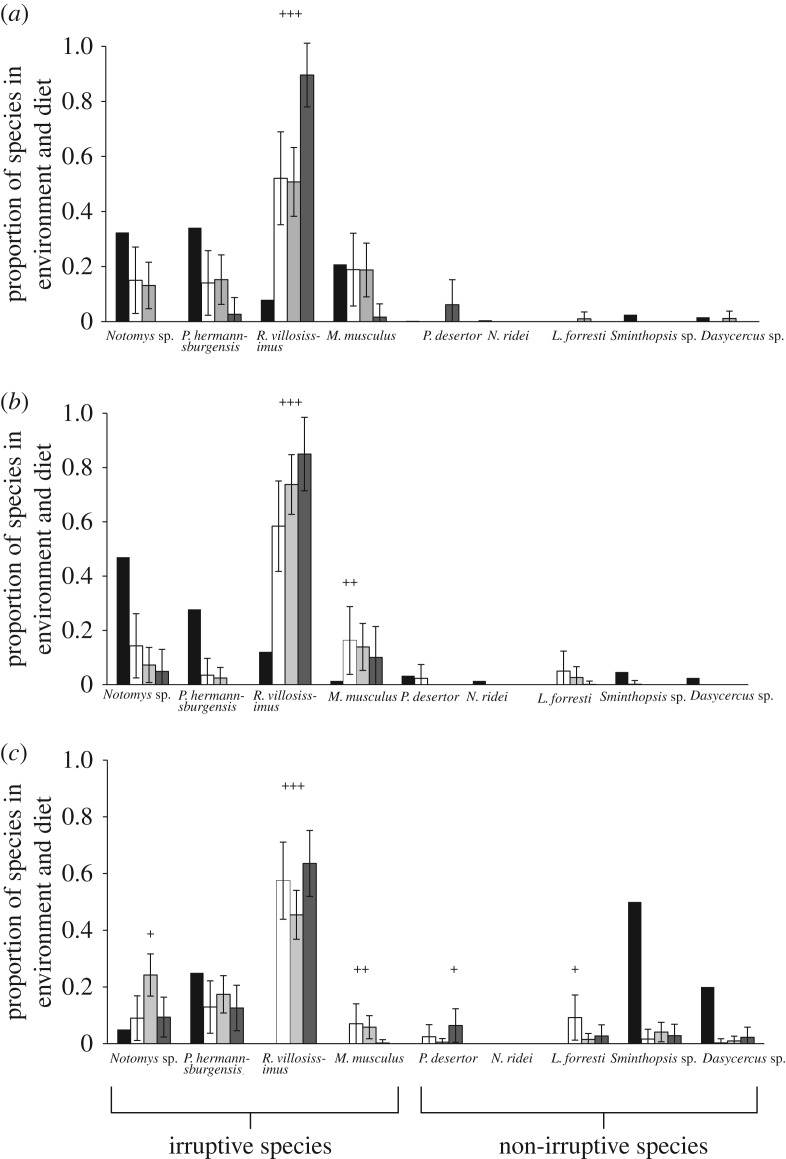
Relative availability of small mammals in the environment over four stages of an irruption cycle in the Simpson Desert, central Australia, and their representation in the diets of the predators. Proportional representation (mean ± 95% CI) of small mammal prey in the diet (based on proportional volumes of prey in scats) of feral cats (white bars), red foxes (light grey bars) and dingo (dark grey bars) and in the environment (based on captures per hectare in traps; black bars), during the (*a*) boom, (*b*) decline and (*c*) early bust stages of a prey irruption cycle in the Simpson Desert, central Australia. Positive selection for specific species is denoted by ‘+’ symbol. Irruptive and non-irruptive small mammals are indicated.

The irruptive small mammals and, in particular, the long-haired rat, comprised the greatest volume of all prey types in each predator's scats across all stages of the prey irruption cycle, but declined during the bust stage in scats of the red fox and dingo (figure 2; see electronic supplementary material, table S2). During the boom, decline and early bust stages, all predators selected for the long-haired rat, but included fewer sandy inland mice and *Sminthopsis* spp. in their diets compared to that which was available in the environment (figure 4). Dasyurids were also generally under-represented in the diets of each predator, across the irruption cycle, as were wongai ningauis (figure 4). The feral cat and red fox showed preference for house mice during the decline and early bust stages (figure 4). All predators selected for spinifex hopping-mice less than their relative abundance in the environment during the boom and decline stages; however, the red fox positively selected this species in the early bust stage (figure 4). During the early bust stage, positive selection was shown by the feral cat for Forrest's mouse (*Leggadina forresti*), and by the dingo for the desert mouse (figure 4).

## 3. Discussion

Our results reveal distinct patterns in the feeding habits of the feral cat, red fox and dingo; however, not all predator species exhibited broad dietary shifts between the high and low stages of the prey irruption cycle as anticipated. In particular, the red fox and dingo changed their diets to include a greater diversity of prey types and fewer small mammals after they declined. The feral cat, conversely, persisted in maintaining a high composition of small mammals in its diet through the irruption cycle, and its dietary diversity hardly altered during the study. Feral cats did, however, display variable use of small mammal species through the irruption cycle. Indeed, all predators altered their selection to include different small mammal species through the irruption cycle, a finding against our initial predictions. We expand upon these findings in more detail below, and comment briefly on the implications of our results for each of the predator species and their prey.

Predators with weak but variable prey preferences typically exhibit greater dietary switching in contrast to those with stronger preferences [31]. It was anticipated that the more opportunistic red fox and dingo would exhibit larger dietary shifts as small mammal populations declined [32,33], in comparison to the feral cat, which often shows much greater preference for particular (mostly smaller) prey types [25,34]. Indeed, more extensive prey shifts have been observed previously in the diets of the red fox and dingo in arid and semi-arid regions, compared to that of the feral cat [35]. The feral cat, however, exhibits moderate dietary flexibility in these systems, including, for example, more reptiles and invertebrates in its diet when fewer small mammals are available [34–36]. These findings stand in contrast to our own; while the feral cat did display some dietary variability and diversity (by including reptiles and invertebrates as dietary components during the early bust stage and birds during the boom and bust stages), it did not significantly vary its diet between the high and low stages of the irruption cycle. This indicates that small mammals probably continued to represent the most energetically profitable meal for the feral cat throughout the study.

The energetic profitability of a prey item may reflect how effective a predator is at capturing and processing that item, and thus could be determined by particular hunting specializations [37]. The feral cat's retractable claws, specialized dentition and sit-and-wait hunting strategy probably provides it with greater success in capturing live prey, allowing it to persist for longer periods on its preferred diet of small mammals [38]. However, while such adaptations might explain why we found so little change in the diet of the feral cat across the irruption cycle in contrast to that of the red fox and dingo, they do not explain why previous work mapping feral cat diets did report increases in dietary breadth after small mammals declined (e.g. [35]). In our system, despite substantial declines in small mammal numbers, the relative densities of alternative prey potentially were not high enough to encourage feral cats to broaden their diets. Indeed, in this arid Australian environment, densities of invertebrates, birds and reptiles during bust stages are typically very low.

Although the feral cat did not exhibit much temporal variation in its diet across the different prey types, it did display greater selection for certain small mammals, especially Forrest's mouse during the post-irruption stage. Increased dietary preferences in this stage were also observed by the red fox, for the spinifex hopping-mouse, and dingo, for the desert mouse. This accords with our expectation that some predators do become more selective as prey populations decline. However, the predators studied had consistently strong preference for the long-haired rat across both the irruption and post-irruption stages. Selection for the long-haired rat probably reflects the species' ease of capture, its preference for open habitats and lack of specific adaptations for escaping predators such as bipedal motion and rapid flight [16]. Further, weighing up to 200 g, this rat was the heaviest small mammal in our system, and thus likely to also meet the higher energy demands required by larger predators. The rat's energetic profitability may therefore explain why, in contrast to studies conducted in systems that did not record long-haired rats among the irrupting species (e.g. [29,35]), the dingo included such a high composition of the small mammal prey type in its diet.

Without measuring additional variables such as predator abundance and the rate of prey consumption, we cannot completely evaluate the effects of the different dietary choices made by predators on their own populations or on specific prey populations. A study that ran concurrently to our own in the Simpson Desert, which recorded predator activity across the 2011 prey irruption using motion sensor cameras does, however, enable us to make some comments on this issue [21]. Greenville *et al*. [21] found a peak in predator activity from June to October 2011, corresponding to the end of the boom stage and the beginning of the early bust stage, after which the numbers of predators observed on cameras dropped to low levels. This suggests the feral cat, red fox and dingo can exhibit a substantial numerical response to declining prey populations, regardless of their different foraging strategies. These predators may rely on other strategies such as nomadic relocation to areas of more abundant prey (e.g. [16]), or otherwise broad-scale movement during times of prey scarcity to ensure ongoing survival (e.g. [39]).

The peak in predator activity recorded across the end of the boom and early bust stages suggests that prey selected during these times might experience high levels of predation pressure [40]. Based on our results, prey most vulnerable includes the long-haired rat during the post-irruptive stage, and the Forrest's mouse, spinifex hopping-mouse and desert mouse, during the early bust stage. While we cannot determine whether predators will contribute to the overall decline of these small mammal populations, increased preference for some of these species may be cause for concern. This is because the feral cat and red fox are efficient introduced predators that have been associated with the decline and extinction of small mammal species in Australia, especially in arid regions [26]. As such, control measures for foxes and cats might have greatest utility during the post-irruptive stages when susceptible prey populations are returning to low levels. To further understand the effects of predators on irruptive prey populations, we encourage long-term monitoring of predator diets across multiple irruption cycles, as well as concurrent tracking of prey and predator abundances. In Australian arid systems, in particular, this should allow us to identify and then mitigate the potential impacts of different predators on vulnerable prey.

## 4. Material and methods

### 4.1. Study system

We conducted our study on Ethabuka Reserve (23°46′ S, 138°28′ E), Simpson Desert, central Australia. The study region is characterized by long, parallel sand dunes that rise to 8–10 m and run in a north/northwest–south/southeast direction [41]. The prevailing habitat is hummock grassland, dominated by spinifex grass (*Triodia basedowii*), although patches of gidgee trees (*Acacia georginae*) are common in the dune swales [42].

The Simpson Desert is a hot desert, although the regional climate is driven by the El Niño Southern Oscillation and thus is highly irregular [18]. There is a pronounced wet season, with most rain falling between December and March; however, annual rainfall is low, with an average of 216.2 mm a year recorded between 1888 and 2011 [13]. Summers are very hot, with mean maximum temperatures over 40°C, and winters cold, with minima below 5°C [41]. During this study, between June 2008 and July 2013, 1410 mm of rain was recorded. Most precipitation was recorded in 2010 and the first three months of 2011, when 968 mm of rain fell in a series of extreme rainfall events.

### 4.2. Scat collection and analysis

Scats were used to quantify feral cat, red fox and dingo diets. Scats were collected along unsealed roads, around water bores, and on dune swales and dune crests, on 16 occasions between June 2008 and July 2013. Most collection sites were searched on each occasion, so scats were not expected to be older than the previous collection period 3–4 months earlier. However, any samples that appeared decomposed or broken were discarded. Scat identification, treatment and sorting followed the methods of Spencer *et al*. [27]. Samples were processed by an independent expert (G. Story, Scats About, Pty, Majors Creek, Australia). For each scat sample, small and large mammalian prey were classified to genus or species, while the remaining prey were identified broadly as ‘medium-sized mammal’, ‘reptile’, ‘bird’, ‘invertebrate’ and ‘vegetation’ prey types. When material could not be identified under any of these categories, it was classified as ‘unknown’.

### 4.3. Estimating prey abundance

To estimate prey abundance, we sampled twenty-eight 1 ha trapping grids, each comprising six lines of six pitfall traps, spaced 0.5–30 km apart throughout the study region. Individual pitfall traps were spaced 20 m apart; the top line of traps was positioned on a dune crest and the bottom line in the swale, so that each grid sampled the topographic range of the dunefield. Traps were opened on 12 or more of these grids four times a year between June 2008 and July 2013 and were checked for three consecutive mornings on each sampling occasion. All captured animals were identified to species and marked by ear-notching in case of recapture. Abundance estimates for each small mammal species were made prior to and during each period when scats were collected. The first trapping session was conducted in June 2008, with the second trapping session and first scat collection following this in September 2008. The last trapping session and scat collection took place in July 2013, so that 17 trapping sessions and 16 scat collections were conducted. All applicable institutional and national guidelines for the care and use of animals were followed. Research was approved by the University of Sydney Animal Ethics Committee (approval L04/4–2009/3/5020).

### 4.4. Data analyses

Based on the small mammal abundance measures, we split the irruption cycle into four stages: ‘late bust’ (July 2008–August 2010), ‘boom’ (September 2010–August 2011), ‘decline’ (September 2011–April 2012), and ‘early bust’ (May 2012–July 2013). These stages roughly follow those adopted in Greenville *et al*. [21]. In particular, the busts are defined by the lowest recorded small mammal abundances and occurred on either side of the irruption. It is likely that we only captured the end of the first bust and the beginning of the second, thus we labelled these stages accordingly. The boom stage was defined as the period in which small mammal densities experienced climbs to their peaks, and the decline stage as when small mammal abundances dropped following the boom stage until the early bust stage.

We grouped prey items in scats into the following categories: small mammals (less than 500 g), medium-sized mammals (501–6999 g), large mammals (≥7000 g), birds, reptiles, arthropods, vegetation, rubbish and other. Because frequency of occurrence may overestimate the importance of small food items [43,44], we calculated the percentage volume of individual prey items and prey types in all predator scats. Volume of each food type or species in scats was expressed as a percentage of the total volume of all food types in the scats, and we used this measure to address the first hypothesis. To assess any differences in predator diets over the four prey-irruption stages, we used a two-way crossed permutation multivariate analysis of variance (PERMANOVA), with all predator species included in the analysis. Irruption stage and predator type were factors (i.e. independent variables) in the PERMANOVA, and we tested for differences in the percentage volumetric composition of all different prey types (response variables) simultaneously. Significant factors (*p* < 0.05) were then identified using pair-wise comparisons. All tests used Bray–Curtis dissimilarity as the metric, and PERMANOVAs were permutated 999 times under a reduced model. We used PRIMER 6 for Windows (v. 6.1.11) for all PERMANOVA analyses [45].

To address the second hypothesis, we plotted the cumulative diversity of prey items identified in each sampling period against the number of scats examined. To calculate diversity we used the Brillouin index, as prey items were sampled non-randomly [46], using the following equation:

$$H=\frac{\ln N!-\ln n_{i}!}{N},$$

where *H* is the dietary diversity of the predator, *N* is the total number of individual prey items recorded, and *n*_i_ is the number of individual prey items of the *i*th type [47]. These results were also used to assess the adequacy of dietary sample sizes for each predator by visualizing whether the diversity of prey items continued to increase as we sampled more scats, as illustrated by the curves reaching an asymptote. To further assess dietary diversity, we calculated diet breadth using Levins' measure of niche breadth [48], standardized on a scale from 0 to 1, using the measure proposed by Hurlbert [49]. Levins' *B* is highest when an equal number of prey types occurs in each resource state, indicating indiscriminant use among resource states, and least when all the prey types occur in only one resource state, indicating maximum specialization [50]. Prey categories (expressed as percentage volume) were used in the calculations, and were produced for each predator over the four stages of the prey-irruption cycle.

For the third hypothesis, we used a Bonferroni simultaneous-confidence-interval approach [51,52] to test for differences between the relative availability of small mammals in the environment over the four stages of the irruption cycle (based on the proportional abundance of individuals captured on the trapping grids) and their representation in the diets of the predators (based on proportional volumetric occurrence of the species in scats). If the proportional availability of a prey category fell above or below the confidence levels estimated for its occurrence in the diet, we concluded that the prey item was avoided (above confidence level) or selected (below confidence level). This approach assumes that the probability of detecting different small mammal species in traps is consistent among species. In our system, prior work has indicated that the relative trappability of many small mammal species accurately reflects their abundances and is not an artefact of changes in animal behaviour or susceptibility to capture [53].

## Supplementary Material

Table S1. Volume composition of prey types in predator scats over the irruption cycle.

## Supplementary Material

Table S2. Small mammals species captured over the irruption cycle.

## Supplementary Material

Figure S1. Small mammals captured over the irruption cycle.

## Supplementary Material

Raw Data

## Data accessibility

The datasets supporting this article are uploaded as electronic supplementary material.
