# Supplementary material for: Prey selection and dietary flexibility of three species of mammalian predator during an irruption of non-cyclic prey
Source: R Soc Open Sci. 2017 Sep 13;4(9):170317. doi: 10.1098/rsos.170317 (PMC5627079; doi:10.1098/rsos.170317)
Supplement: Table S1. Volume composition of prey types in predator scats over the irruption cycle. [file rsos170317supp1.pdf]

Table S1. Percentage volumetric composition of prey types in feral cat (*Felis catus*), red fox (*Vulpes vulpes*) and dingo (*Canis dingo*) scats, grouped across four different stages of an irruption and collected in the Simpson Desert, central Australia.

	**Cat**				**Fox**				**Dingo**			
	**Late Bust**	**Boom**	**Decline**	**Early Bust**	**Late Bust**	**Boom**	**Decline**	**Early Bust**	**Late Bust**	**Boom**	**Decline**	**Early Bust**
**Large Mammal**	**0**	**0**	**0**	**0**	**0**	**0**	**0**	**1**	**30**	**6**	**0**	**23**
Macropus/Osphranter sp.	0	0	0	0	0	0	0	1	30	6	0	22
Other large mammals	0	0	0	0	0	0	0	0	0	0	0	1
**Small Mammal**	**79**	**75**	**87**	**70**	**64**	**93**	**97**	**36**	**42**	**82**	**90**	**32**
**Muridae sp.**	**68**	**72**	**87**	**64**	**42**	**92**	**96**	**33**	**42**	**82**	**90**	**29**
Notomys sp.	3	11	12	6	15	12	7	9	2	0	4	3
Pseudomys hermannsburgensis	38	10	3	8	16	14	2	6	30	2	0	4
Pseudomys desertor	0	0	2	1	0	0	0	0	0	5	0	2
Leggadina forresti	0	0	4	4	0	1	3	1	0	0	0	1
Mus musculus	25	14	14	5	6	17	13	2	10	1	9	0
Rattus villosissimus	3	38	51	39	5	47	71	16	0	73	76	20
Ningaui ridei	0	0	0	0	0	0	0	0	0	0	0	0
Dasycercus sp.	7	0	0	0	17	1	0	0	0	0	0	1
Sminthopsis sp.	3	0	0	1	4	0	0	1	0	0	0	1
Unidentified small mammal	1	3	0	5	0	0	0	1	0	0	0	1
**Medium Mammal**	**4**	**0**	**0**	**2**	**1**	**0**	**0**	**1**	**0**	**6**	**0**	**8**
**Reptile**	**5**	**3**	**7**	**10**	**7**	**1**	**0**	**7**	**19**	**0**	**3**	**17**
**Bird**	**7**	**14**	**2**	**12**	**8**	**1**	**0**	**8**	**1**	**1**	**0**	**9**
**Invertebrates**	**2**	**4**	**3**	**5**	**12**	**2**	**0**	**16**	**3**	**1**	**4**	**4**
**Vegetation**	**0**	**0**	**0**	**2**	**8**	**2**	**2**	**29**	**4**	**6**	**3**	**7**
**Unknown**	**3**	**4**	**1**	**0**	**2**	**0**	**0**	**0**	**2**	**0**	**<1**	**<1**
Number of scats	*24*	*57*	*69*	*104*	*34*	*150*	*126*	*262*	*10*	*36*	*55*	*135*

Note: due to rounding errors totals do not always add to exactly 100.
