# Supplementary material for: Prey selection and dietary flexibility of three species of mammalian predator during an irruption of non-cyclic prey
Source: R Soc Open Sci. 2017 Sep 13;4(9):170317. doi: 10.1098/rsos.170317 (PMC5627079; doi:10.1098/rsos.170317)
Supplement: Table S2. Small mammals species captured over the irruption cycle. [file rsos170317supp2.pdf]

Table S2. Small mammals captured over the course of the study between June 2008 and July 2013 (n = 3820 captures), expressed as numbers of captures per 100 trap nights (trap night = 1 trap open for one night). Captures of small mammals are shown separately for each species, for each stage of the irruption cycle.

	Dasycercus cristicauda	*Mus musculus*	*Ningaui ridei*	*Notomys alexis*	*Pseudomys desertor*	*Pseudomys hermannsburgensis*	*Rattus villosissimus*	*Sminthopsis hirtipes*	*Sminthopsis macroura*	*Sminthopsis youngsoni*	Unknown rodent	Total small mammals
**Late Bust**	0.56	0.07	0.18	1.40	0.00	3.33	0.00	0.57	0.00	1.04	0.00	7.15
**Boom**	1.21	19.85	0.18	25.37	0.18	30.59	6.54	0.73	0.00	1.28	0.22	85.89
**Decline**	0.67	0.70	0.48	12.11	0.44	5.81	2.85	0.33	0.07	0.48	0.04	23.96
**Early Bust**	0.09	0.00	0.00	0.43	0.05	0.59	0.00	0.05	0.00	0.31	0.00	1.52
**Total**	2.53	20.62	0.84	39.31	0.67	40.33	9.39	1.68	0.07	3.11	0.26	118.52
