# Supplementary material for: Prey selection and dietary flexibility of three species of mammalian predator during an irruption of non-cyclic prey
Source: R Soc Open Sci. 2017 Sep 13;4(9):170317. doi: 10.1098/rsos.170317 (PMC5627079; doi:10.1098/rsos.170317)

Figure S1. Small mammals captured over the course of the study between June 2008 and July 2013 (n = 3820 captures)

Expressed as numbers of captures per 100 trap nights (trap night = 1 trap open for one night). Captures of all species of small mammals are aggregated.

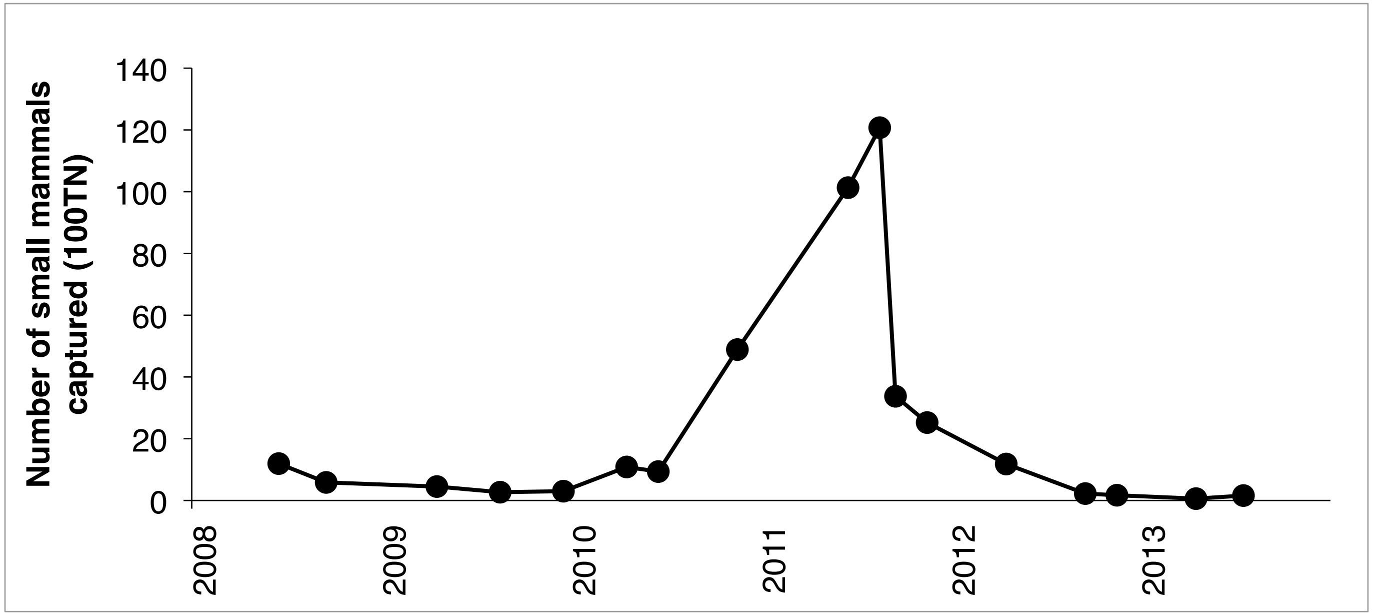

Supplement: Figure S1. Small mammals captured over the irruption cycle. [file rsos170317supp3.pdf]
